# The roles of metabolic profiles and intracellular signaling pathways of tumor microenvironment cells in angiogenesis of solid tumors

**DOI:** 10.1186/s12964-022-00951-y

**Published:** 2022-11-23

**Authors:** Hamidreza Zalpoor, Fatemeh Aziziyan, Mahsa Liaghat, Maryam Bakhtiyari, Abdullatif Akbari, Mohsen Nabi-Afjadi, Razieh Forghaniesfidvajani, Nima Rezaei

**Affiliations:** 1 Shiraz Neuroscience Research Center, Shiraz University of Medical Sciences, Shiraz, Iran; 2 Network of Immunity in Infection, Malignancy & Autoimmunity (NIIMA), Universal Scientific Education & Research Network (USERN), Tehran, Iran; 3 Department of Biochemistry, Faculty of Biological Sciences, Tarbiat Modares University, Tehran, Iran; 4 Department of Medical Laboratory Sciences, Faculty of Medical Sciences, Islamic Azad University, Kazerun Branch, Kazerun, Iran; 5 Department of Medical Laboratory Sciences, Faculty of Allied Medicine, Qazvin University of Medical Sciences, Qazvin, Iran; 6 Research Center for Immunodeficiencies, Children’s Medical Center, Tehran University of Medical Sciences, Dr. Gharib St, Keshavarz Blvd, Tehran, Iran; 7 Department of Immunology, School of Medicine, Tehran University of Medical Sciences, Tehran, Iran

**Keywords:** Tumor microenvironment, Solid tumors, Angiogenesis, Metabolic profile, Signaling pathways, Cancer therapy

## Abstract

Innate and adaptive immune cells patrol and survey throughout the human body and sometimes reside in the tumor microenvironment (TME) with a variety of cell types and nutrients that may differ from those in which they developed. The metabolic pathways and metabolites of immune cells are rooted in cell physiology, and not only provide nutrients and energy for cell growth and survival but also influencing cell differentiation and effector functions. Nowadays, there is a growing awareness that metabolic processes occurring in cancer cells can affect immune cell function and lead to tumor immune evasion and angiogenesis. In order to safely treat cancer patients and prevent immune checkpoint blockade-induced toxicities and autoimmunity, we suggest using anti-angiogenic drugs solely or combined with Immune checkpoint blockers (ICBs) to boost the safety and effectiveness of cancer therapy. As a consequence, there is significant and escalating attention to discovering techniques that target metabolism as a new method of cancer therapy. In this review, a summary of immune-metabolic processes and their potential role in the stimulation of intracellular signaling in TME cells that lead to tumor angiogenesis, and therapeutic applications is provided.

**Video abstract**

**Supplementary Information:**

The online version contains supplementary material available at 10.1186/s12964-022-00951-y.

## Introduction

Both developed and developing nations continue to be heavily burdened economically and socially by cancer. There are expected to be 19.3 million new cancer cases worldwide in 2020 (excluding nonmelanoma skin cancer) as well as almost 10 million deaths due to cancer (9.9 million excluding nonmelanoma skin cancer) [1]. Therefore, understanding how cancer develops and progresses is imperative for developing interventions aimed at promoting the well-being of cancer patients [2, 3]. So, in order to better understand cancer, we should review the literature on the tumor microenvironment (TME) [4–6]. In this line, metabolic modifications and physiological processes play a crucial role in the development of resistance to immune checkpoint inhibitors (ICIs) in cancerous cells [7]. Fermentation/anaerobic glycolysis or "Warburg effect" is one of these metabolism modifications producing anabolic precursors, such as lactate, required by rapidly dividing embryonic tissues and tumors despite its low ATP yield/glucose molecule. Also, increased ketone bodies, branched-chain amino acids, and other toxic metabolites produced due to dysfunction of key enzymes of TCA cycle called “oncometabolites” affect the TME cells to promote angiogenesis and tumor growth as well as hypoxia [8]. In response to hypoxia, hypoxia-inducible factor-1 (HIF-1) alpha stabilizes the vascular endothelial growth factor (VEGF) A promoter and activates its gene expression [9]. Upregulated VEGF and numerous signaling pathways activated by HIF-1 promote angiogenesis and tumor growth through several strategies. Moreover, increased lactate levels by upregulating lactate dehydrogenase (LDH) and monocarboxylate transporters (MCTs) via anaerobic glycolysis, acidifying the TME facilitating angiogenesis, tumor growth, and drug resistance through several mechanisms and signaling pathways [10]. So, understanding these TME-modified metabolic pathways and other modifications, such as hypoxia, in TME and solid tumor cells could be beneficial for finding new and potential therapeutic strategies that will be discussed in this review.

### Tumor angiogenesis process

Some diseases including cancer, diabetic retinopathy, and rheumatoid arthritis are associated with angiogenesis, regardless of physiological conditions [11]. Angiogenesis is related to tumor growth and metastasis [12]. There are several steps in Angiogenesis, such as the separation of endothelial cells from pericytes and the basement membrane, invasion and migration across the basement membrane, and, finally, an extension of the angiogenesis into the tumor [13]. In this regard, several factors induce angiogenesis, including VEGF, angiopoietins, transforming growth factors (TGF), platelet-derived growth factor (PDGF), tumor necrosis factor-α (TNF-α), interleukins, and the members of the fibroblast growth factor (FGF) family [14, 15]. Of these, the tumor-secreted cytokine VEGF family has a crucial role in both normal and tumor-induced angiogenesis [16]. VEGF A, B, C, and E can interact with VEGF receptor-1 (VEGFR-1), VEGFR-2 on vascular endothelial cells (ECs) and neurons. Hematopoietic stem cells (HSCs), monocytes, and osteoblasts can also be stimulated by VEGF-A. It can also induce the production of nitric oxide (NO) causing vasodilation [17, 18]. Additionally, Angiopoietin-2 (Ang-2) is a downstream target of VEGF signaling [19]. It should be mentioned that NO and Ang-2 act as angiogenic switches [19, 20].

Moreover, in response to the activation of VEGFR1, different signaling pathways are activated, including phosphoinositide-3-kinase (PI3K)/protein kinase B (PKB/Akt), mitogen-activated protein kinase (MAPK)/extracellular signal-regulated kinase (ERK) pathway (p38-MAPK/ERK1/2) [21, 22] facilitating the migration of inflammatory cells, the release of inflammatory cytokines, and the release of proteolytic enzymes into the extracellular matrix (ECM) [23, 24]. As a result, endothelial cells are proliferated through activation of phospholipase-Cγ (PLCγ)/protein kinase C (PKC) and Ras/ Raf/ERK/MAPK due to the binding of VEGF to the extracellular domain of VEGFR-2 [21, 25–27].

Ang-2 attaches to Tie-2 receptors on endothelial and leukemia cells [28–30]. The receptor activates Src homology 2 containing tyrosine phosphatase protein (SHP2), growth factor receptor-bound protein 7 (GRB7), and focal adhesion kinase (FAK) which in turn promotes cell survival and migration [31]. On another hand, competition between Ang-1 and Ang-2 for the Tie-2 receptor causes Ang-1-mediated stabilization to be blocked by Ang-2 [32].

VEGF-A, Ang-2, and MMPs also, begin to destabilize pre-existing capillaries, which prepares the capillaries for sprouting endothelial cells. The tips of the vessels are endothelial cells that sprout filopodia-like extensions from the primary vessel. Two cell surface proteins called Delta-like 4 (Dll4) and Notch with its ligands, regulate the number and activity of the cells in the tip [33]. So, the Notch/Dll4 pathway is responsible for regulating vessel sprouting and maturation. In response to increasing the Dll4, the basic fibroblast growth factor (FGF), VEGF-A, hepatocyte growth factor (HGF) and VEGFR-1 levels increase [34].

At last, in order for a vessel to function properly, it must be stabilized, and this requires endothelial cell-to-cell contact or interactions between endothelial cells and pericytes. This interaction is promoted by Ang-1 and PDGF-A, -B, -C, -D [9]. In turn, tumors overexpressing Ang-1 and PDGF fail to promote blood vessel maturation as well as resist the effects of antiangiogenic treatment and chemotherapy. Although, the data suggest that tumor microenvironments contain more destabilizing vascular factors than Ang-1 and PDGF [35]. Notably, Endothelial cells secrete a large amount of Ang-2 in response to hypoxia or VEGF-A, which prevents the normalization of blood vessels. In a study, inhibiting Ang-2 and VEGF synergically increased pericyte coverage, VE-cadherin tight junction, and decreased permeability [36]. MMPs are also one of the factors that exert their destabilizing activity through pericyte detachment, cell–cell adhesion cleavage, and degradation of ECM [37].

## Innate and adaptive immune cells in TME that lead to tumor angiogenesis

**Fig. 1:**
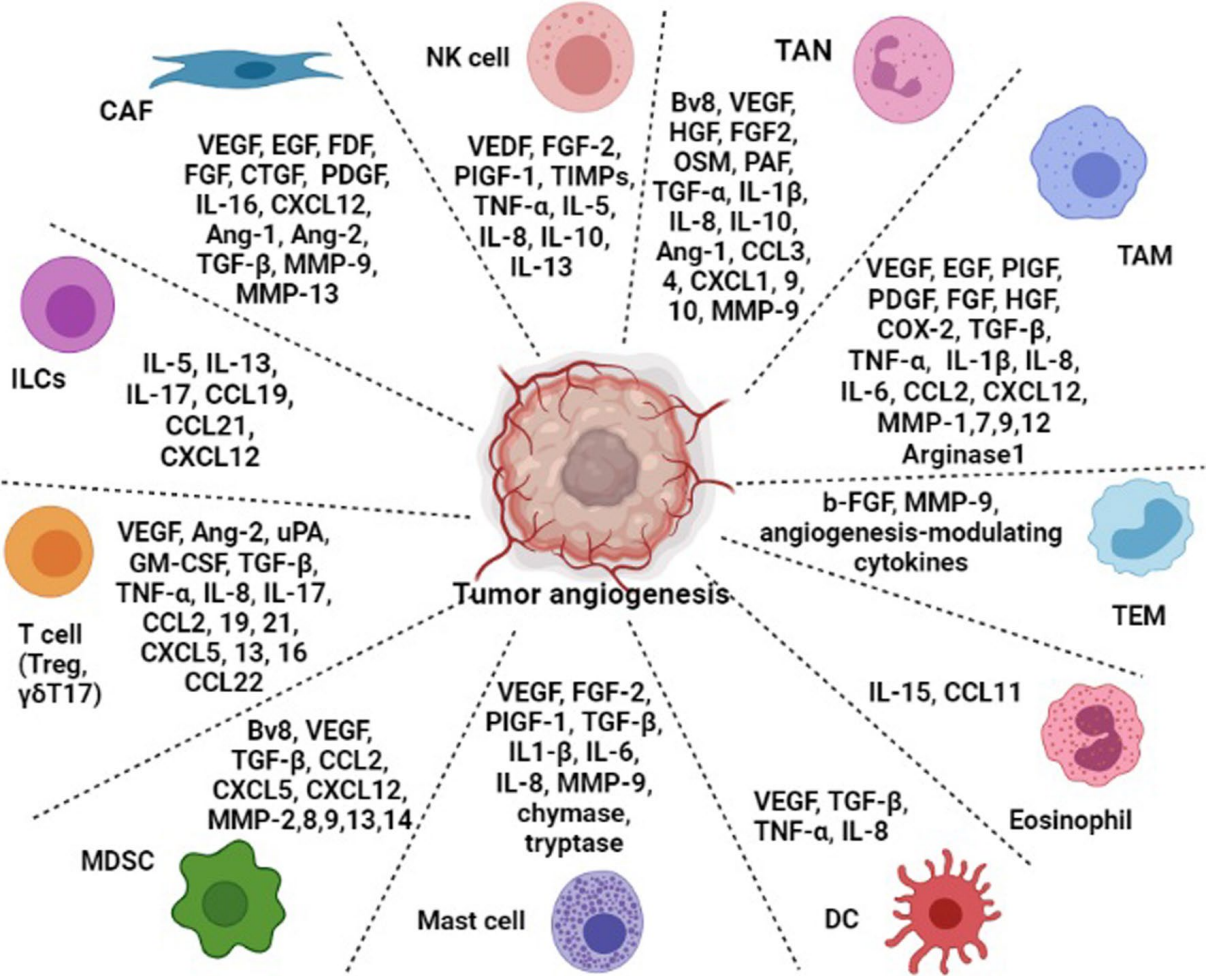
Contribution of innate and adaptive immune cells in tumor microenvironment to tumor angiogenesis. Within the tumor microenvironment, soluble mediators (cytokines, chemokines, and enzymes) exert their role directly as proangiogenic factors expressed by M2-like tumor-associated macrophage (TAM), myeloid-derived-suppressor cell (MDSC), N2-like tumor-associated neutrophil (TAN), natural killer (NK) cells, mast cells, and dendritic cell (DC), cancer-associated fibroblast (CAF), Tie2-expressing monocytes (TEM), eosinophil, Innate lymphoid cells (ILCs), and T cells (regulatory T cell, γδT17)

There are many mechanisms that contribute to tumor angiogenesis and immunosuppression in TME [38]. Several laboratory studies have demonstrated that stromal cells, such as fibroblasts and myeloid cells, can promote tumor angiogenesis via expressing various pro-angiogenic factors, such as Bv8/PROK2, members of the VEGF, FGF, PDGF, and angiopoietin families [39–44]. TME myeloid cells produce increased fatty acid synthase in response to CSF1, which leads to the expression of PPARβ/δ-dependent genes, such as VEGF, arginase1 (Arg1), and IL-10 contributing to angiogenesis and immunosuppression [45]. Tumor-associated macrophage (TAM) and tumor-associated neutrophil (TAN) have pro-tumor activities through extracellular matrix (ECM) remodeling, enhanced invasion and metastasis of cancer cells, angiogenesis, cancer cell proliferation, lymphangiogenesis, and inhibition of anti-tumor immune surveillance [46]. Therefore, the investigation of TME cells is crucial to our understanding of tumor angiogenesis, and to enhancing the effectiveness of cancer therapy (Fig. 1.).

### Natural killer (NK) cells

According to the pro-tumorigenic phenotype of cancer infiltrating NK cells, they can secrete pro-angiogenic factors, such as VEGF, transforming growth factor-beta (TGF-β), IL-8, IL-10, placental growth factor (PlGF), Ang-1, and Ang-2 [47]. The most relevant seems to be VEGF. Accordingly, a loss of HIF-1α in NK cells increased the bioavailability of VEGF by reducing the infiltration of NK cells through the VEGF receptor-1 (VEGFR-1) [48]. Additionally, zoledronic acid enhances NK cell activity on VEGF by synergizing with IL-2 [49]. With regard to the reports, in non-small cell lung cancers (NSCLC), the levels of VEGF, IL-8, and PlGF synthesized by NK cells are higher than in controls [50]. In a study of colorectal cancer patients, Bruno et al. [50] demonstrated that NK cells express matrix metalloproteinase-2 (MMP-2), and tissue inhibitor of MMP (TIMP), and angiogenin. Additionally, STAT3/STAT5 activation has been found in tumor-associated NK cells (TANKs) [51], and treatment with a STAT5 inhibitor called pimozide reduced the ability of endothelial cells to produce VEGF [51].

### Dendritic cells (DCs)

Dendritic cells (DCs) are generally divided into two subgroups: myeloid DC (MDCs) and plasmacytoid DC (PDCs). MDCs within the bone marrow are immature DCs with high phagocytic capability. MDCs, also called conventional DCs (cDCs), are composed of a number of distinct subsets of cells with potent antigen-presenting capacities [52], playing a crucial role in the activation of T cell responses in response to pathogens and tumor cells [53]. Despite this, DCs in the TME containing tumor-associated cDCs or regulatory DCs (regDCs) exhibit altered functions with impaired cross-presentation capacity, express low levels of co-stimulatory molecules, as well as have high-proangiogenic activity. During tumor progression, these changes depend on various conditions, such as hypoxia, production of PGE2, IL-10, adenosine, and lactate level increase [54]. There is evidence that soluble factors derived from tumor cells may interfere with this maturation process and hinder the development of mature DCs [55]. The accumulation of immature DC in tumors is consistent with this, as only very few mature MDC are observed in tumors [55]. Immature DC can be recruited to the TME by tumor-derived factors such as VEGF, HGF, b-defensin, CXCL8, and CXCL12 [55, 56]. Angiogenic cytokines released by tumor-associated DCs, including VEGF, TGF-β, TNF-α, IL-8, and osteopontin, directly contribute to tumor angiogenesis [54, 56, 57]. According to the studies, tumors could attract PDCs to enhance angiogenesis while excluding MDCs to inhibit angiogenesis, demonstrating a novel mechanism for modulating tumor neovascularization [56]. Thus, blocking tumor-associated DCs that stimulate angiogenesis in TME may be a potential strategy in cancer therapy.

### Mast cells (MCs)

According to the mast cells (MCs) pro-tumorigenic phenotype, they produce several pro-angiogenic factors, such as VEGF, FGF-2, TGF-β, IL-8, TNF-α, and nerve growth factor (NGF). A correlation between MCs and VEGF with angiogenesis has been shown in laryngeal squamous cell carcinoma and in lung cancer [58–61]. Moreover, as part of angiogenic factors stored in mast cell granules, two proteases, namely tryptase and chymase, play an important role in the angiogenic responses of MCs [62]. Tryptase stimulates endothelial cell proliferation, degrades connective tissue matrix, promotes vascular tube formation in vitro, and activates matrix metalloproteinases (MMPs) and plasminogen activator (PA), degrading the extracellular matrix, thereby releasing FGF-2 or VEGF from matrix-bound cells [62]. Several hematological and solid tumors, such as breast cancer [63], melanoma, colon-rectal cancer, uterine cervix cancer, and pulmonary adenocarcinoma [64], as well as vascular tumors, such as haemangioma and haemangioblastoma, have been shown to have an increased number of MCs associated with angiogenesis. Hence, the accumulation of MCs leads to an increase in neovascularization, mast cell VEGF and FGF-2 expression, tumor aggressiveness, and poor prognosis [62]. Also, mast cells produce TIMPs, which have a role in the regulation of extracellular matrix degradation, allowing the secretion of angiogenic factors [62].

### Myeloid-derived suppressor cells (MDSC)

The immune-suppressive TME causes the appearance of the phenotype and functional alterations of various players, such as Myeloid-derived suppressor cells (MDSCs). MDSCs are capable of invading directly into the tumor endothelium. They also secrete many pro-angiogenic factors. In TME, MDSCs are able to infiltrate tumor tissues and also differentiate into TAM [65]. In addition, MDSCs have a key role in the production of MMPs, chemoattractants, and the creation of pre-metastatic environments that contribute to cancer invasion, metastasis, and angiogenesis [65]. MDSCs can lead to the induction of an immune-suppressive environment [66] and angiogenesis directly as well as indirectly (by interacting with several components of innate and adaptive immunity) [67]. MDSCs are capable of boosting angiogenesis and promoting tumor neovasculature, through the production of high levels of MMPs, such as MMP2, MMP8, MMP9, MMP13, and MMP14. Additionally, MDSCs can also stimulate tumor growth and blood vessel growth [67, 68]. As a result of a recent study, it has demonstrated that MDSCs with the production of high levels of MMP9 stimulate VEGF function through boosting its bioavailability [69]. As part of the regulation of angiogenesis, Bombina variegate peptide 8 (Bv8) and vascular endothelial growth factor (EG-VEGF), and TGF-β are the key molecules. Of these, Bv8 is also capable of recruiting MDSCs to tumor tissues [54, 70]. Recruitment of MDSCs is mediated by chemokines and chemoattractants. Most solid tumors show necrotic regions induced by hypoxia. HIF-1α (hypoxia-inducible factor 1α), as a transcription factor, is expressed under hypoxic conditions. HIF1α overexpression can trigger tumor cells to secrete chemoattractants, including stromal-derived factor 1α (SDF-1α or CXCL12), CXCL5, and CCL2. To recruit MDSCs, these ligands could bind to the receptors on MDSCs [65]. Also, Sorrentino et al. and Iannone et al. observed that MDSCs triggered adenosine receptor A2B-induced VEGF production, vessel density, and angiogenic activity [71, 72]. Moreover, MDSCs have crosstalk with NK cells. MDSCs can also inhibit the anti-tumor responses of NK cells, increase angiogenesis [67], establish pre-metastatic niches [73], and recruit other immunosuppressive cells [74]. It has been found that MDSCs significantly reduce NK cell cytotoxicity in breast cancer, resulting in an increase in metastatic potential [75]. High ROS levels (radical oxygen species), created in cancerous conditions, exert a crucial role in stimulating the MDSCs and VEGF receptor expression on the MDSCs and their expansion in the TME [67].

### Tumor associated macrophages (TAM)

The most frequent immune cells of the tumor microenvironment (TME) are tumor-associated macrophages (TAMs). The macrophages displayed different phenotypes based on the microenvironment they populated. The two main types of activated macrophages are classical activated macrophages (M1) and alternatively activated macrophages (M2) [76]. Macrophages of type M1 promote inflammatory responses against pathogens and tumor cells, while macrophages of type M2 have anti-inflammatory properties that promote wound healing and tumor progression [77]. Increasing evidence points to the fact that TAMs contribute to tumor progression via multiple mechanisms. TAMs have been found to release pro-angiogenic factors, such as VEGF-A, EGF, PlGF, PDGF, FGF, HGF, TGF-β, TNF-α, IL-1β, IL-8, CCL2, CXCL8, and CXCL12 [54, 76–78]. TAMs contribute to tumor progression within the TME through cross-talking with other leukocytes, inflammatory and stromal cells. Also, TAMs can directly recruit T regulatory cells (Treg) by secreting CCL20 and CCL22 chemokines and they can activate them by secreting IL-10 and TGF-β [54]. On the other hand, several transcription factors are involved in M1/M2 polarization: NF-κB, STAT1, and IRF5 involved in M1 polarization, whereas MYC, STAT6, KLF4, IRF4, and PPARγ are associated with M2 polarization [79]. Studies have shown that hypoxic TME polarizes macrophages into the M2 phenotype and TAMs have a significant role in inducing tumor angiogenesis [80]. In this line, expression of HIF-1α in TAMs induces VEGF-A production [77]. Werno et al. revealed that HIF-1α expression in macrophages plays a key role in tumor angiogenesis when breast cancer cells are co-cultured with wild-type or HIF-1α knockout macrophages [81]. Through the production of growth factors, chemokines, and cytokines, TAMs produce an immunosuppressive TME that inhibits anti-tumor responses [77]. Moreover, they function as angiogenesis promoters by the production of pro-angiogenic factors and MMPs such as MMP-1, MMP-7, MMP-9, and MMP-12, as well as vascular construction which supplies nutrients and oxygen to solid tumor cells [54]. Additionally, an ovarian cancer mouse model found that TAMs were major sources of MMP-9, and MMP9-producing TAMs, were positively related to tumor angiogenesis and tumor growth [82]. Through the production of pro-inflammatory mediators, such as EGFR family ligands, TNF-α, IL-6, IFN-γ, proteases, reactive oxygen species (ROS), and nitrogen species, TAMs create a mutagenic microenvironment. It has been observed that microvascular density correlates positively with TAMs and VEGF levels in mammary tumors [83]. Vascular neuropilin-1 (NRP-1) has been shown to be a variable receptor for two secreted glycoproteins, VEGF-A and Semaphorin 3A (Sema3A), but also its role as an adhesion receptor is poorly understood [84, 85]. As a response to Sema3A, NRP-1 plays a key role in the entry of TAMs into hypoxic niches, but the loss of NRP-1 promotes anti-tumor immunity and hinders angiogenesis [77]. In cervical cancer, NRP-1 plays a crucial role in hypoxic TME-induced activation and TAM-induced pro-tumoral effects [80]. According to biochemical studies, NRP-1 might have a role in VEGF-mediated induction of ERK, Akt, P38 MAPK, SRC, and p130 CAS pathways [84, 86]. So, NRP-1 may play roles in angiogenesis, which likely synergize with its known status as a co-receptor for VEGFR2 [84].

Quaranta et al. found that TAMs have a significant role in tumor-associated fibroblasts (TAFs) activation for producing an excessive amount of extracellular matrix (ECM), by secreting granulin [87]. Moreover, releasing urokinase-type plasminogen activator (UPA) and thymidine phosphorylase (TP) by TAMs stimulates tumor angiogenesis by increasing endothelial cells (ECs) migration, degradation of ECM, as well as a vascular invasion [88]. The M2 macrophages, also known as alternatively activated macrophages, can be further classified into M2a, M2b, M2c, and M2d. Through adenosine, the M2d phenotype can be induced in pro-inflammatory M1 macrophages via adenosine 2A receptor (A2AR) activation [89]. Indirectly IL-17 triggers differentiation of M2 macrophages through stimulation of the COX-2/PGE2 pathway in cancer cells [90]. Additionally, the studies suggest that FGF signaling may contribute to M2a-induced angiogenesis and PlGF signaling to M2c-induced angiogenesis, but that more research should clarify the exact mechanisms involved [91]. Therefore, M2d macrophage can produce VEGF and VEGF receptors [92] indicating its pro-angiogenic phenotype.

### Tumor-associated neutrophils (TANs)

In the TME, it has been reported that macrophages and fibroblasts promote the growth of colorectal cancer. While neutrophils were originally thought to have defensive functions, it has been shown that some populations of neutrophils, called tumor-associated neutrophils (TANs), are cancer-supportive via TGF-β and interferon-β signaling controlling the plasticity between tumor-supportive and tumor-suppressive neutrophils [93].

TANs play an important role in tumor metastasis and angiogenesis due to their ability to release a variety of proangiogenic and immunosuppressive factors such as VEGF, IL-1β, TGF-α, FGF2, HGF, and Ang-1; as well as chemokines including CXCL1, CXCL10, CXCL9, CXCL8, CCL4, and CCL3; and enzymes involved in ECM remodeling (MMP-9) [54].

G-CSF (CSF3) and its receptor CSF3R are required for neutrophil proliferation and growth. STAT3, which is downstream of an activated CSF3R, is important for cancer inflammation. Neutrophils increase the expression of BV8 (prokineticin-2) via CSF3, which causes myeloid cell mobility and myeloid-dependent tumor angiogenesis. The activation of STAT3 is required for the synthesis of BV8 [54]. In addition, G-CSF, IL-6, VEGF, and IL1-β are among the cytokines produced by tumor and stromal cells that cause neutrophilia and make these neutrophils more suppressive. TANs are thought to circulate longer than other circulating neutrophils. Interactions between TANs and neoplastic cells result in the release of GM-CSF by the tumor cells. GM-CSF stimulates the release of Oncostatin M (OSM) and neutrophil synthesis. OSM is a member of the IL-6 family of cytokines that can enhance VEGF production via the Jak/STAT pathway [94].

It is believed that reactive oxygen species (ROS), reactive nitrogen species (RNS), and proteases released by neutrophils are involved in tumor initiation [95]. In tumor cells, neutrophil elastase (NE) can inhibit insulin receptor substrate-1 (IRS-1). A decrease in IRS-1 levels can enhance the interaction between PI3K and PDGF-R, a factor that promotes tumor cell proliferation [96]. In addition, through cyclooxygenase-2 (COX-2)-mediated prostaglandin E2 (PGE2) synthesis, it also increases tumor cell growth [97].

TANs also play a role in tumor invasion and angiogenesis in primary and metastatic sites by generating MMP9, VEGF, HGF, PAF, IL-10 [54, 93]. Modulating MMP-9 enhances angiogenesis by activating VEGF. It is reported that neutrophil-derived tissue inhibitors of metalloproteinase (TIMP)-free MMP-9 induce angiogenesis strongly [98]. These MMP-9 s generated by tumor-infiltrating neutrophils regulate tumor cell invasion and tumor angiogenesis at the same time [98]. Neutrophils also aid tumor cell spreading by trapping circulating tumor cells with neutrophil extracellular traps and enhancing their migration to distant locations [93].

Another factor is IL-8, which is a multifunctional cytokine secreted by neutrophils after cell activation [99]. Studies have shown IL-8 signaling promotes angiogenic responses in endothelial cells, increases the proliferation and survival of both cancer and endothelial cells, and stimulates the migration of the cancer cells, endothelial cells, and neutrophils [100]. A major mechanism of IL-8's biological effects is its binding to two cell-surface G protein-coupled receptors called CXCR1 and CXCR2 [101, 102]. Several studies have shown tumor cells overexpress IL-8 in response to chemotherapy or environmental stress, such as hypoxia. Given the presence of CXCR1 and CXCR2 receptors on endothelial cells, cancer cells, and TANs, increased IL-8 secretion from tumor cells has a broader significance for the TME [103].

Another factor that plays a vital role in TME modification and angiogenesis is HGF. HGF acts as a cell adhesion complex and indirectly increases the production of IL-8 and VEGF. The HGF role in cancer growth can be regulated by several signaling pathways, including the PI3K and MAPK pathways. Besides that, HGF also regulates metastasis and invasion [94].

So, according to the TANs' role in tumor metastasis and angiogenesis, some therapeutic methods targeting TANs were suggested, with two basic approaches: (a) targeting the CXCL-8/CXCR-1/CXCR-2 axis to block TANs, or (b) targeting substances released by polymorpho-nuclear cells that stimulate cancer growth [104].

### Carcinoma-associated fibroblasts (CAFs)

Fibroblasts are primarily responsible for the synthesis of the extracellular matrix (ECM) and are not epithelial, vascular, or hematopoietic cells [105]. Although angiogenesis, ECM remodeling, and epithelial proliferation are adaptive for healing wounds, within the tumor microenvironment, they promote tumor growth and development [106].

In research by Orimo et al., fibroblasts were isolated from human breast carcinomas and normal breast fibroblasts were isolated from the same person. The fibroblasts were co-injected into nude mice with breast carcinoma cells, and carcinoma-associated fibroblasts (CAFs) significantly grew tumors more than normal fibroblasts did. It has been shown that this is due to the high levels of CXCL12 secreted by CAFs, which recruit endothelial progenitors to tumors and increase vascularization [107]. In addition, Yang et al. showed that CAFs from human prostate cancers also incited xenograft growth via connective tissue growth factor (CTGF). In the xenograft model, CTGF expression proved to be induced by TGF-β, and overexpression of CTGF in 3T3 fibroblasts led to enhanced microvessel density and tumor growth [108]. CAFs can also contribute to angiogenesis indirectly by releasing active growth factors from the ECM when they express MMPs. In this line, CAFs produce MMP-9 and MMP-13, both involved in angiogenesis. There is evidence that both MMP-9 and MMP-13 increase angiogenesis in tumors by releasing VEGF from the ECM [106]. Tumor vascularization was decreased in integrin α1 knock-out mice that lack integrin α1β1, a blocker of MMP synthesis. This was due to the increased production of angiostatin [109]. MMP-7 and MMP-9 also, act on circulating plasminogen to produce angiostatin. So, these factors indicate that MMPs play contradictory roles in angiogenesis [110].

In addition, CAFs produce more IL-6 than fibroblasts in normal tissues preventing tumor cell apoptosis through a STAT3-dependent mechanism [111] and enhancing angiogenesis [112].

Furthermore, HGFs are expressed by CAFs and they play a key role in angiogenesis. The CAFs produce angiogenic factors, such as VEGF, TGF-β1, EGF, Ang-1, Ang-2, PDGF, MMPs, and FDF, which are essential for hepatocellular carcinoma (HCC) initiation, progression, and metastatic development, as well as the growth of new vessels. When CAFs are activated, the VEGF receptor, the PDGF receptor, and the Tie-2 receptor are upregulated, which results in increased mitogenesis through VEGF [113]. Hormonal stimulation such as leptin, or physical stress like hypoxia, is known to induce VEGF secretion by hepatic stellate cells (HSCs) and has been found to be upregulated in HCC [113]. HCC cells' conditioned medium can activate CAFs and promote VEGF production by activating the Akt-VEGF pathway and subsequently, increasing the oxidative stress in hepatocellular carcinomas (HCC) promoting their potential malignancy [114].

Most believe that there is a strong link between tumor angiogenesis and notch signaling cascades, FGF, VEGF, and angiopoietin (ANGPT). Pro-angiogenic FGF2 stimulates proliferation and migration of endothelial cells directly through activation of FGFR1 (or FGFR2) as well as indirectly by inducing VEGF and ANGPT2 from endothelial cells. ANGPT1 is made by pericytes, and it induces Tie2 signaling, which controls endothelial quiescence or stability. ANGPT2 is secreted by endothelial cells and inhibits Tie2 signaling to promote endothelial activation and growth [115]. Dll4 expression is induced by VEGF signaling in endothelial tip cells, which then activates Notch signaling in endothelial stalk cells for vascular inactivation via downregulation of VEGFR [116]. In endothelial activation, VEGF, FGF2, and ANGPT2 participate, while ANGPT1 and Notch contribute to inactivity. To stimulate tumor angiogenesis in endothelial cells, the VEGFR2 and FGFR1/2 are two crucial receptors as tyrosine kinases (RTKs) [116]. So, a monoclonal antibody (mAb) or small-molecule VEGFR inhibitor is commonly used to target VEGF signaling in cancer patients. But some tumors fail to respond to the VEGF blockade treatment and others recur after it is stopped. One of the major mechanisms responsible for VEGF blockade therapy resistance is the activation of FGF signaling in endothelial cells [115]. Therefore, FGFR inhibitors may be effective in overcoming resistance to this therapy. Two options are available to block the dual signal cascade of FGF and VEGF. It is better to use monotherapy using small-molecule FGFR/VEGFR2 dual inhibitors such as AZD4547 and dovitinib in order to reduce medical costs, but combination therapy using anti-VEGF mAb and FGFR inhibitors may be better to prevent adverse effects. In order to optimize FGF/VEGF dual blockade therapy, safety issues, and medical costs must be considered [116]. While in the context of anti-angiogenic therapy targeting the VEGF pathway, there are adverse effects such as hypertension, bleeding, and thrombosis [117]. Multi-kinase inhibitors such as AZD4547, dovitinib, and ponatinib also, target FGFRs and other tyrosine kinases. Selective FGFR targeting is intended to lessen side effects, whereas the dual targeting of VEGFR/CSF1R and FGFR is predicted to increase anti-tumor effects indirectly by normalizing the TME [116].

### Innate lymphoid cells (ILC)

The innate lymphoid cell (ILC), found mostly in solid tissues, is a family of mononuclear hematopoietic cells [54]. ILC family members include NK cells, Group-1 ILCs (ILC1), Group-2 ILCs (ILC2), Group-3 ILCs (ILC3), and lymphoid tissue inducer cells (LTis) [118, 119]. ILCs exhibit high cell plasticity and can easily be converted into different subtypes after exposure to TME stimuli [54]. Moreover, ILC1 can promote tumorigenesis when it is converted into NCR (NKp46, NKp44)^+^ ILC3 [120].

It is still debated whether ILCs play a role in cancer progression or prevention [54]. ILC2 releases type 2-cytokines such as IL-5 and IL-13; both stimulate angiogenesis [54]. By secreting IL-22, ILC3s support epithelial stability and maintain tissue homeostasis. ILC3s are known to produce IL-17 and CXCL12, which play a role in tumorigenesis, angiogenesis, and tumor growth. There is increasing evidence to suggest that ILC3s play a role in recruiting Treg cells and MDSCs to TME and promoting M2-like macrophages there [54]. CCL21 was used in a syngeneic 4T1.2 mouse breast model to attract ILC3s to primary tumors, which then induces tumor stromal cells to secrete CXCL13, and then results in lymphotoxin and activates receptor of NF-κB ligands, which stimulate tumor cell migration and lymphangiogenesis [121]. Breast cancer patients with invasive behavior also show an association with genes expressed by ILC3, such as CXCL13, CCL21, CCL19, CCR7, and CXCR5. Researchers have shown that ILC3 creates the tertiary lymphoid structures (TLS), which are responsible for tumor growth and lymph node metastasis. On the other hand, the tumor-preventing and tumor-promoting effects of TLS remain contested [54]. In inflamed tissue from patients who suffer from chronic obstructive pulmonary disease or smokers, NRP-1^+^ LTi-like ILC3s have been detected, which were associated with VEGF production [122]. Immunohistochemical studies of inflamed tissues indicated that RORγτ^+^NRP-1^+^ cells were associated with blood vessels as well as in the alveolar parenchyma, showing their role in angiogenesis and triggering of lung TLS. Aside from IL-22 and IL-17, the pro-inflammatory LTi-like NRP-1^+^ ILC3 subgroup was discovered to produce CSF2, TNF-α, B-cell activating factor, and CXCL8, all of which might lead to angiogenesis [54].

Thus, while the abilities of ILC3 may encourage tumor growth, neoangiogenesis, epithelial-mesenchymal transition, and metastasis; others may instead promote anti-tumor responses [120]. Researchers have also studied the effects of ILCs on tumor vessels in cancer suppression mediated by cytokines, such as IL-12 [123, 124]. It has been shown that IL-12 inhibits angiogenesis by interacting with NK cells in lymphomas [125]. IL-12-activated NK cells have the ability to cause endothelial cell cytotoxicity in vitro, which reduces tumor angiogenesis [125]. There is evidence that other populations of ILCs mediate IL-12's antitumor activity in melanoma. Eisenring et al. discovered that a group of IL-12-driven NKp46^+^ ILC3s causes overexpression of the adhesion molecules ICAM and VCAM, resulting in enhanced leukocyte infiltration and tumor control [123]. Tumor-infiltrating natural cytotoxicity receptor (NCR)^+^ ILC3 cells in NSCLC tissues also induced upregulation of these adhesion molecules [126]. Additionally, IL-17 produced by ILC3s may influence tumor vasculature. As a matter of fact, IL-17 stimulates angiogenic factors in stromal cells, including VEGF, TGF-β, and IL-8 [127]. Additionally, IL-17 increased blood vessel permeability and E-Selectin and VCAM-1 expression in lung endothelial cells, resulting in pulmonary metastasis [128]. So, a targeted strategy has yet to be developed for non-NK ILCs because of their recent discovery and incomplete understanding of their role in tumor growth and angiogenesis [54].

### Eosinophils

Eosinophils characterization is the expression of CCR3 and CD125. Eosinophils have been found to increase in several human tumors, including gastrointestinal tumors, oral squamous cell carcinoma, nasopharyngeal carcinoma, and Hodgkin lymphoma [129]. In specimens of non-small cell lung carcinoma (NSCLC), the association between eosinophil density and angiogenesis was demonstrated, as well as their relationship to tumor stage [130]. It has been demonstrated that CCL11 (eotaxin), a highly potent and specific eosinophil chemoattractant, via binding to CCR3 is responsible for attracting eosinophils to the TME [131]. Additionally, eosinophil recruitment at tumor sites may lead to angiogenesis, because the secretory granules of eosinophils contain VEGF, which is rapidly secreted when activated by IL-15 [129]. However, the exact role exerted by eosinophils in the TME remains controversial.

### Tie2-expressing monocytes (TEM)

As opposed to other monocyte populations, the newly discovered Tie2-expressing monocyte (TEM) expresses the Tie2 receptor for angiopoietin, an attribute that is unique to this population [132, 133] and various human tumor entities have been reported to contain TEM [134].

In this regard, angiopoietin-2 (ANGPT2), as the Tie2 ligand, plays an important role in regulating TEM recruitment [133, 134]. Tumor-infiltrating TEM has been demonstrated to accumulate in close proximity to blood vessels and to hypoxic areas of tumors [134, 135]. By localizing TEM near tumor blood vessels, sanctions would potentially affect the process of tumor angiogenesis. Moreover, studies show that selective removal of TEM from the TME significantly reduced angiogenesis and impaired glioma growth [135]. Interestingly, in spite of the fact that TEM numbers are lower than TAM and granulocytes within the tumor, TEM has a significant role in contributing to vessel neoformation, which suggests that TEM is a potent driver of tumor angiogenesis [135]. In recent studies, it has been found that TEM frequency correlated with angiogenesis in tumor tissues and may serve as a diagnostic marker for NSCLC [136], glioblastoma [137], HCC [138], and Renal Cell Carcinoma (RCC) [139]. In addition, Tie2 and VEGFR pathways play varying roles in TEM angiogenic and lymphangiogenic activities across breast cancer (BC) patients; nevertheless, a combination of Tie2 and VEGFR kinase inhibitors inhibited these activities and overcame inter-patient variability [140]. The expression of MMP-9, b-FGF and angiogenesis-modulating cytokines are the critical factors in transmitting angiogenic signals by TEM [55, 135]. Although the mechanisms of how TEM stimulates angiogenesis are still under debate, current studies are studying the effects of TEM on tumor angiogenesis.

### Regulatory T cells (Tregs)

It is well known that regulatory T cells (Tregs) play an important role in tumor progression and tumor angiogenesis, as they are highly enriched in the TME [38]. A number of factors may contribute to the increased number of Tregs at tumor sites [38]. The environment of tumors such as ovarian cancer and Hodgkin lymphoma contains high concentrations of CC-chemokine ligand 22 (CCL22), which is secreted from both tumor macrophages and tumor cells. Through CCR4, CCL22 recruits Tregs, and Treg migration is inhibited through CCR4 blockade in vitro [38]. Tregs in hypoxic areas are capable of stimulating angiogenesis via VEGF production. As well as VEGF, other angiogenic factors produced by Tregs are Leptin and NRP-1 [141]. The expression of NRP-1 on Treg cells correlates with Foxp3 expression and suppressor function in vitro. NRP-1 was found to promote angiogenesis via interaction with VEGF-A165 (and other VEGFs), and VEGF-R2 enhances signaling through this pathway [141]. Moreover, Tregs indirectly promote angiogenesis by blocking the angiostatic cytokines IFN-γ and CXCL-10 released by effector cells [38]. So, VEGFR2 plays a key role in tumor angiogenesis, and it revealed that this receptor expresses on the surface of Tregs [142].

### IL-17-producing T cells: γδT and Th17

The evidence suggests that IL-17 is a key cytokine that plays a significant role in various inflammatory diseases, as well as tumorigenesis [143]. A variety of T-cell subsets produce IL-17, including CD8^+^ T cells, CD4^+^ T cells, NKT cells and γδT cells [143–145]. Th17 exerts its role by several transcription factors, including HIF1α, RORγt, RORα, IRF4, AHR, c-Rel, IκBζ, BATF, and RUNX1 [146]. Also, γδT cells have various transcription factors, such as RORγt, RelB, RUNX1, AHR, and Hes1 [146]. IL-17-producing γσT cells are tumor-promoting cells that induce angiogenesis in response to the TME [145]. Also according to studies, Th17 cells and IL-17 have been reported to promote anti-VEGF therapy resistance by recruiting immunosuppressive and proangiogenic myeloid cells to the TME [43, 147]. The expression of IL-17 in tumor microenvironments is well established [148, 149]. Studies revealed that as a result of the IL-17 produced by IL-17-producing γδT cells, VEGF, Ang-2, GM-CSF, IL-8, and other angiogenesis factors productions were induced [54]. However, it also stimulates the production of anti-angiogenic factors such as thrombospondin-1 (TSP1), TIMP1, serpine-1, and platelet factor 4 [54]. Furthermore, angiogenesis may be accelerated by the accumulation of other intratumoral IL-17-producing cells, such as Th17 [150]. According to a recent study, IL-17-producing γδT cells homing to inflamed skin depend on CCR6. However, it is not clear precisely how IL-17 influences tumor development [151]. By doing so, a potential cancer immunotherapy approach would be to manipulate the production of IL-17, and other proangiogenic factors by human γδT and Th17 cells, as well as CCR6, or factors involved in γδT17 cell proangiogenic polarization.

## Metabolic alterations of cancer cells

Cancer cells are assumed to need to reprogram their catabolic and anabolic metabolism for energy intake and biomass synthesis for cell survival and development in order to initiate and progress, especially in unfavorable microenvironmental conditions [152–154]. Otto Warburg discovered nearly a century ago that cancer cells used glucose a lot through aerobic glycolysis [155]. Based on investigations in the field over the last two decades have not only proved that oncogenic defects are mostly responsible for the Warburg Effect in cancer cells, but they have also indicated that metabolic reprogramming has developed considerably beyond what was initially expected [154]. Given the increasing importance of this dysregulated metabolism in cancer biology, it seems appropriate to review what we know about cancer metabolic reprogramming other than the Warburg effect by addressing the following main points. How do cancer cells manage their anabolic metabolism to support rapid proliferation? What are the other energy sources besides the main ones like glucose and glutamine used by cancer cells? How can cancer cells use metabolic reprogramming to communicate with and guide their microenvironment? Because glycolysis and the tricarboxylic acid cycle occur in cells, glucose and glutamine are two major nutritional sources for cancer cell survival and growth (TCA). Conventional waste products from cells, such as lactate, ketone bodies, acetate, ammonia, and other foreign proteins, have long been considered useless metabolites in association with these processes. Remarkably, advances in recent years have described a variety of new features in cancer cells for those conventional waste products, which have gradually evolved into unconventional nutrient sources for ATP production and biomass synthesis for essential components during cancer cells' reaction to stressed stated. We mentioned these unusual nutrient functions in tumor progression bellow.

In recent studies, several typical waste products have been recognized as unconventional nutrient sources, including lactate, ketone bodies, acetate, ammonia, and exogenous proteins.

1. Lactate produced by lactate dehydrogenase A (LDHA) can be exported to the extracellular environment by monocarboxylate transporter 4 (MCT4) in particular; exogenous lactate imported by monocarboxylate transporter 1 (MCT1) can be converted to pyruvate by LDHA in the cytosol or lactate dehydrogenase B (LDHB) in the mitochondria while simultaneously reducing NAD^+^ to NADH to enter the TCA cycle. Lactate dehydrogenase (LDH), which is commonly activated by oncoproteins like cMyc, HIF-1α, and mTOR in cancer cells, converts pyruvate to lactate while simultaneously oxidizing NADH to NAD^+^ [156–159].

For many years, lactate was supposed to be a normal byproduct of cellular metabolism. But recent research indicates that lactate behaves as a complex immunomodulatory component that regulates the activity or functions of the innate and adaptive immune systems. The innate and adaptive immune responses in the intestine and other systemic regions are thus shaped by lactate, a crucial new signaling molecule. In addition, lactate's pleiotropic effects modulate several immune cell functions in the microenvironment and pathological cases [160–162].

Excessive lactate released by reprogramming cancer cells' metabolism affects immunological responses through extracellular acidification, serving as an energy source by migrating among various cell populations and blocking the mTOR pathway in immune cells [163, 164].

Exogenous lactate increases the migration and invasion of cancer cells in a concentration-dependent approach using the Boyden chamber assay [165], stimulates various oncogenic signaling pathways [166], and positively correlates with radioresistance [167]. Lactate has also been found to acidify the tumor microenvironment and modify numerous immune cells, allowing them to evade immune surveillance and radiotherapy resistance in cancerous patients [168].

2. Acetate is absorbed and converted to cytosolic acetyl-CoA by cytoplasmic acetyl-CoA synthetase 2 (ACSS2), which recent research suggests that acetate potentially plays an essential role in sterol, cholesterol synthesis, and fatty acid production and histone acetylation, particularly in hypoxic and low-lipid [169].

ACSS2 has also been identified as a critical enzyme in the production of acetyl-CoA from acetate, and its expression has been linked to tumor aggressiveness in various organs. These results point to the possibility that acetate use is a common characteristic of many tumors [170].

**Fig. 2:**
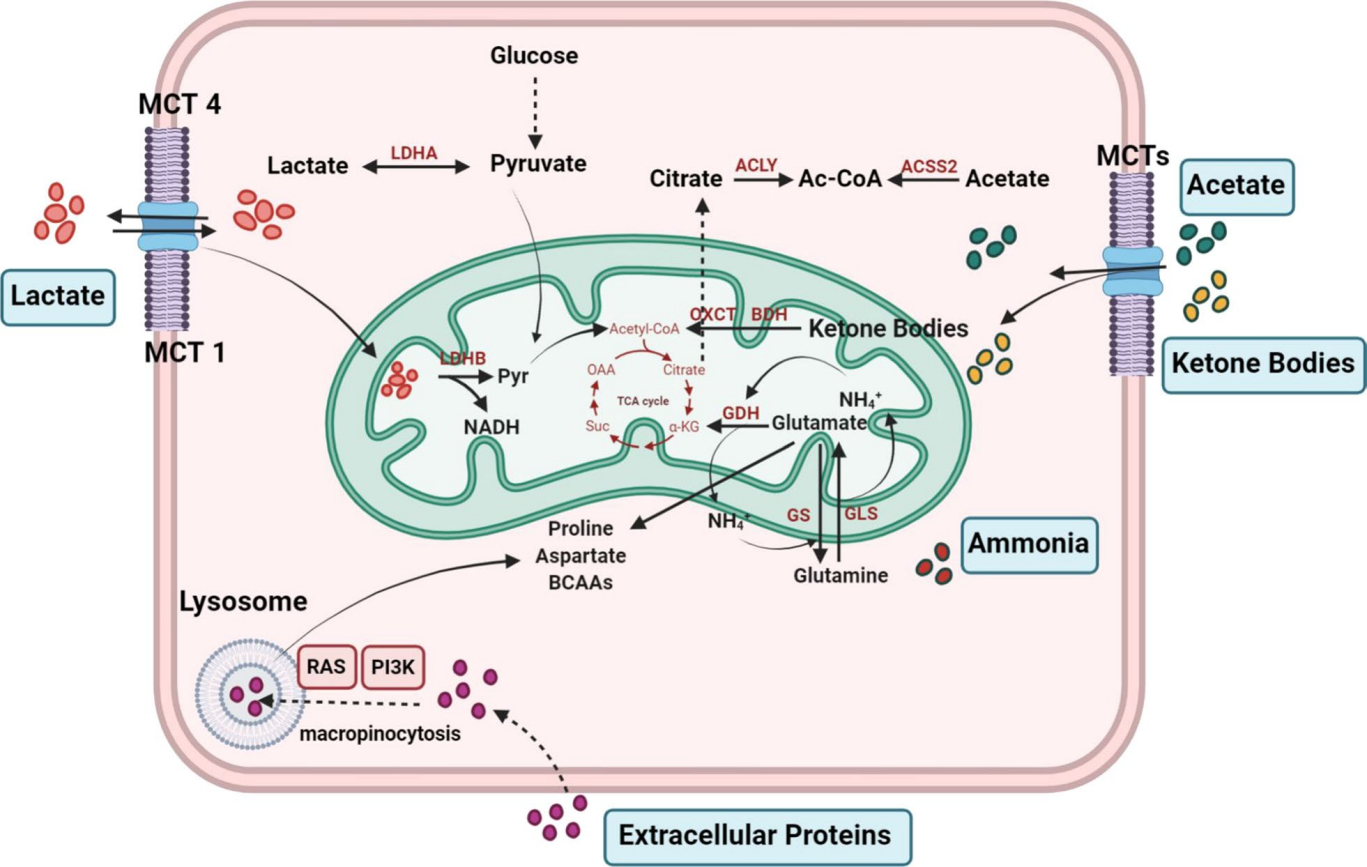
Interactions between metabolism and the tumor microenvironment. The chemical properties of the extracellular space are altered by cancer cells, which has complex effects on the characteristics of normal cells in the tumor microenvironments, as well as the extracellular matrix. In addition, the cancer cells; metabolic and signaling responses are influenced by the microenvironment

3. In mitochondria, enzymes such as D-OHB dehydrogenase (BDH) and succinyl-CoA: 3-oxoacid-CoA transferase (OXCT) induce ketolysis (ketone body catabolism), To restore the acetyl-CoA pool. Studies have identified a surprising relationship between ketolysis and liver cancer progression, suggesting novel targets for liver cancer therapy based on its pathological mechanism for liver cancer [171].
4. Ammonia generated by glutaminase in the primary organ can be used by glutamate dehydrogenase in the secondary organ to produce glutamate or α-ketoglutarate under certain conditions. The production of proline, aspartate, and BCAAs (branched-chain amino acids) is facilitated by increased glutamate levels. Because of deficient vascularization, ammonia frequently concentrates in the tumor microenvironment, leading to its repletion into cancer cells. The destination and functions of the ammonia produced by cancer cells remain unknown. It would be better sense if the ammonia could be utilized in a metabolic pathway by the tumor cells, as it may be a nitrogen donor [172].
5. The RAS and PI3K pathways enable cancer cells to grow and survive. In this regard, under the control of RAS and PI3K, extracellular proteins can be absorbed, digested, and degraded into amino acids in tumor cell lysosomes, a process known as macropinocytosis. Macropinocytosis is a conserved endosomal process in lysosomes that utilizes extracellular protein degradation to free amino acids [154] (Fig. 2).

### The effects of metabolic alterations in cancer cells and TME cells on each other

One of the important risk factors in cancer angiogenesis is the metabolic alteration of cancer cells. So, targeting key metabolic enzymes and/or mitochondrial metabolic pathways in the hypoxic conditions of the TME, can be a valuable and new anti-cancer therapy [173–175].

Information about a cell's metabolism has the ability to influence not just the cell's own programming, but also the destiny of other cells in its proximity. However, a range of genetically stable cell types, including endothelial cells, TAFs, and innate and adaptive immune system components, have been shown to develop phenotypic alterations as a result of living close to developing tumors [4]. Although it is unclear how cancer cells reprogram their microenvironment to promote tumor growth and dissemination, it is apparent that certain reprogramming involves a variety of mechanisms, such as secreted growth factors, changes in cell–cell interactions, and the extracellular matrix play a crucial role in this line. So, proliferating cancer cells affect the metabolic content of the extracellular environment around them. For instance, extracellular lactate accumulates as a result of cancer cell's excessive use of extracellular glucose and glutamine, which has been reported to influence a variety of cell types in tumor microenvironments. High lactate levels, which inhibit monocyte migration and dendritic and T cell activation, promote the formation of immune-permissive microenvironments [165, 176, 177]. Lactate also causes local macrophages to polarize into the so-called M2 state, which is essential in immunosuppression and wound healing [178, 179]. Moreover, lactate accumulation is important in the stimulation of angiogenesis. Lactate increases HIF-1α stability and VEGF release from tumor-associated stromal cells, and also activation of NF-κB and PI3K signaling in endothelial cells [180–183]. Lactate stimulates the formation of hyaluronic acid by fibroblasts, which may promote tumor invasion [184]. In hypoxia conditions, lactate secretion into the extracellular environment via the monocarboxylate transporter MCT1 is associated with H^+^ co-transport, causing the cell microenvironment to become acidic. Also, the excess CO2 produced during mitochondrial decarboxylation pathways increases extracellular acidity. CO2 diffuses into the extracellular environment, where it is converted to H^+^ and HCO3 by an extracellular type of carbonic anhydrases [185]. So, during this condition, the expression of carbonic anhydrases, particularly the CAIX isoform, is upregulated through hyper-activated HIF-1. In the following, the proteolytic activity of MMPs and cathepsins are promoted by greater extracellular acidity, enhancing the breakdown of extracellular matrix proteins and increasing tumor invasion. Although lactate accumulation and extracellular acidification may be considered a side effect of cancer-specific metabolic reprogramming. On the other hand, ROS produced by the tumor induces oxidative stress in the cancer adjacent fibroblasts leading to a reduction in their mitochondrial function and increase of glucose uptake, which facilitate metabolic reprogramming and differentiation into CAFs. CAFs, as neighborhood cells of tumor cells representing a significant portion of the tumor mass, have quite similarly metabolic reprogramming relative to the tumor cells. CAFs produce high-energy metabolites, such as lactate, pyruvate, and ketone bodies that fuel the neighboring tumor cells. So, these acidic products, particularly lactic acid, acidify TME, as an important stimulator for tumor progression angiogenesis [186–190].

**Fig. 3:**
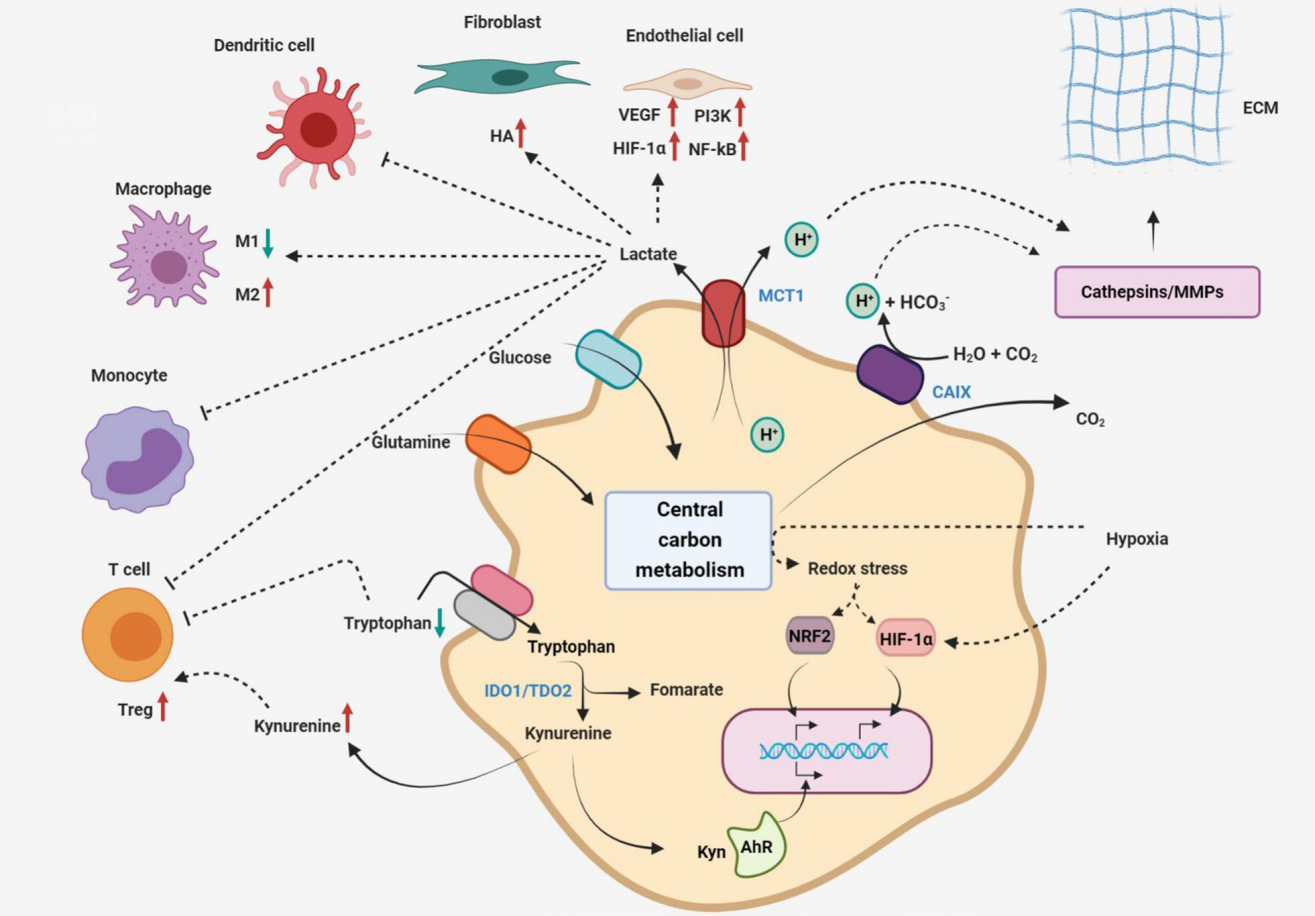
Links between metabolism and the microenvironment. Cancer cells change the extracellular milieu's chemical composition, which has pleiotropic effects on the phenotypes of cells around the tumor and the extracellular matrix. The microenvironment influences the metabolic and signaling responses of cancer cells reciprocally. MMPs: matrix metalloproteinases, AhR: aryl -hydrocarbon receptor, HA, hyaluronic acid, MCT1: monocarboxylate transporter 1, Kyn, kynurenine, TDO2: tryptophan-2, 3-dioxygenase 2, IDO1: indoleamine-2, 3-dioxygenase 1, CAIX: carbonic anhydrase IX, Treg: regulatory T cells, ECM: extracellular matrix

Various tumors use a different process to stimulate the development of an immune-permissive microenvironment around them. Especially, the tryptophan-degrading dioxygenases indoleamine-2, 3- ioxygenase (IDO1), and tryptophan-2, 3-dioxygenase (TDO2), which catalyze the converting tryptophan, into its derivative, kynurenine, are overexpressed in a variety of solid tumor types [191]. As a result, tryptophan deficiency induces effector T cells death as a result of amino acid starvation [192]. Additionally, kynurenine accumulates can act as a ligand for aryl hydrocarbon receptors (AhR) [193]. Kynurenine enhances the regulatory T-cell phenotype in a mechanism that is dependent on AhR, contributing to the inhibition of anti-tumor immune responses [194]. Finally, kynurenine stimulates extracellular matrix breakdown and invasion by influencing autocrine signaling through AhR on cancer cells [193]. Clinical trials for small molecule inhibitors of IDO1 are presently underway [195]. In addition, the conditions in the TME have a significant impact on a cancer cell's metabolism. Tumors are constantly faced with nutrient and oxygen-depleted environments and they adopt a variety of nutrient-scavenging mechanisms to overcome these constraint conditions. Hypoxia affects cell's ability to carry out oxidative phosphorylation and other oxygen-dependent activities, affecting the redox balance and altering cellular signaling and transcriptional pathways. On the other hand, a disorder in the TCA cycle of cancerous cells through loss-of-function mutations in its enzymes such as succinate dehydrogenase, fumarate hydratase and isocitrate dehydrogenase cause to toxic accumulation of succinate, fumarate and L-2-hydroxyglutarate and D-2-hydroxyglutarat called Oncometabolites. These Oncometabolites contribute to angiogenesis and cancer cell growth by altering the expression of the related genes required for malignant features such as HIF-1 [187, 196–198]. Other metabolites such as lysophosphatidic acid (LPA) and adenosine, accumulated in the extracellular milieu of the TME, also promote tumor growth by affecting the immune functions. In cancer cells, an excessive amount of synthesized LPAs are released into the TME. On the other hand, some TME cells, like Cancer-associated adipocytes (CAAs) and TAMs, also contribute to increase the LPA into the TME. Increased LPA of TME induces aerobic glycolysis in the cancerous cells, stimulates pro-tumorigenic features of TAMs, suppresses immune activity of T lymphocytes and subsequently, promotes cancer cell proliferation and migration [199–202]. Adenosine is also, abundantly produced by the tumor as well as by cells in the TME such as CAFs and immune cells infiltrating the area by the action of the ecto-enzymes of CD39 and CD73 overexpressed to catabolize the ATP. Adenosine riched TME directly leads to tumor growth by binding the adenosines to four distinct GPCRs. Moreover, an adenosine rich TME has immunosuppressive effects through reducing cytotoxic activities of T lymphocytes and natural killer (NK) cells and reduced capacity of neutrophils to phagocytose, degranulate, adhere to endothelial cells and produce ROS and inhibiting functions of Tregs [187, 203–205]. Interestingly, some metabolic enzymes, such as fructose-bisphosphatase 1 (FBP1), pyruvate kinase M2 (PKM2) and malate dehydrogenase 1 (MDH1), act as a tumor suppressor or oncogenic factor alongside their canonical role. For instance, FBP1 enzyme inhibits Notch signaling in breast cancer by HIF-1 un-stabilizing and regulating the Wnt/β-catenin pathway. So, FBP1 enzyme plays a tumor suppressive role downregulated in tumor cells. On the other hand, PKM2 and MDH1 enzymes act as oncogenic factors. In this line, PKM2 enzyme interacts with anti-apoptotic protein Bcl-2 increasing its stability and tumor cells' survival. PKM2 enzyme also increases the activity of STAT3 and HIF-1α and their downstream factors/genes promoting angiogenesis and tumor growth. To support the cancerous cells survival, MDH1 enzyme directly binds to p53 increasing its stability and transcriptional activity [206–210]. Hence, taken together, reciprocal interactions among cancer cells and their microenvironments generate a selective influence on cancer cell metabolism, promoting the development of a more aggressive state (Fig. 3)**.**

## Induced tumor angiogenesis by stimulated signaling pathways

Obviously, signaling pathways play a key role in angiogenesis. A similar orchestra leads to tumor expansion. Hypoxia results from insufficient blood supply in the TME area and lead to activation of HIF-1 and upregulation of VEGF [211, 212]. HIF-1 consists of HIF-1α that is sensitive to oxygen pressure unlike HIF-1β (structural subunit). In normoxia, HIF-1α is hydroxylated with Poly Hydroxylase Domain protein (PHD), the Von Hippel-Lindau protein (pVHL) recognizes hydroxylated HIF-1α and links to Elogin C and musters ubiquitin ligase complex, eventually, proteasome degrades hydroxylated HIF-1α after polyubiquitination [213]. Inevitably, a significant amount of redox changes are always seen in the TME that lead to stability of HIF-1α. For instance, increased amounts of ROS inhibit PHD activity through iron oxidation, or NO targets the pVHL oxygen-dependent region commonly known as S-Nitrosylation, and prevents HIF-1α detection by pVHL [214]. According to studies PHD needs α-KG to hydroxylate HIF-1α, but there is a condition called pseudohypoxia that is caused by substances such as 2-hydroxyglutarate (2-HG). 2-HG due to very strong competition with α-KG prevents α-KG connection to PHD and inhibits it [215].

In absence of oxygen, PI3K/Akt/mTOR pathway causes activation of HIF-1α, Importantly Akt as a serine/tyrosine contributes to the activation of mTOR [208, 216, 217]. According to studies by Carbonneau, M. et al. in addition to preventing PHD activity, 2-HG also activates mTOR as a transcriptional factor (3) [218]. Likewise, activated HIF-1α gets dimerized in the nucleus with HIF-1β and forms HIF-1. Binding this heterodimer to the hypoxia response element (HRE) and adjoining to CBP/P300 in the promoter area leads to rising above transcription of VGEF genes [219, 220].

On surface of Tip cells as a leader with numerous filopodia have been expressed VEGFR2, VEGFR3 in a vast number and interaction with VEGF and these receptors lead to activation of the promoter of VEGFR2 and delta-like ligand 4 (Dll4) [221–223]. Dll4 is a type of notch ligand that has a high expression in Tip cells, on the other hand, its notch receptor is expressed on another type of EC that is called stalk cells. Interestingly, engagement of Dll4/notch cleavages notch intracellular domain (NICD), likewise, NICD translocates into the nucleus and links with Rbpj/Cbf1 transcription factors results in gene expressions that are involved in differentiation and proliferation of cells [224, 225]. Moreover, this signaling pathway leads to a decrease of VEGFR2, VEGFR3 expression and an increase of VEGFR1 expression which finally prevents stalk cells from turning into Tip cells [221].

In order for new vessels to form a vascular network, Tip cells are able to link each other with filopodia [226], but the role of myeloid cells such as Microglial and macrophage shouldn't be overlooked. Macrophages under the influence of Ang-2 secreted from ECs migrate forward Tip cells [226]. Importantly it is essential that both Tip cells in junction site turn into stalk cells. Macrophages are the main leaders in this event, and secrete VEGF that affects VEGFR3 on Tip cells, the downstream signaling pathway activates FoxC2 transcription factor that advances gene transcription in favor of converting Tip to stalk [227]. According to studies by Zonneville et al. TGF-β causes Smad3/4 signaling pathway in fibroblast cells via interaction with TGFRB1. This signaling pathway increases fibronectin production and deposition. Interestingly fibronectin, not only strengthens the pericytes and ECs bond but also holds PDGF in TME in favor of pericytes signaling pathways [228]. PDGF-β that is secreted from ECs effects on PDGFBR-β and causes proliferation and migration of pericytes [229]. Migration of pericytes is up to Ras/Raf/MAPK signaling pathway that recruits cellular cytoskeleton in favor of migration. PI3K/PKC/TGF-β and PI3K/Akt/NF-κB signaling pathways both are involved in the proliferation of pericytes [230].

In vacuolization VEGF is the radical factor and as mentioned hypoxia has an effect on VEGF production directly. According to the studies, mTOR has a crucial role in angiogenesis [231]. For instance, VEGF and VEGFR2 connection results Y951 site autophosphorylation in VEGFR2 that results in TSAd/Src/PI3K/Akt/mTOR signaling pathway and significantly increases cell survival [25, 232–234]. Y1214 tyrosine residue actives NCK/FYN/PAK2/CDC42/P38MAPK signaling pathway and at the end P38MAPK causes migration by the use of cellular fibers [235]. RAS/RAF/ERK signaling pathway which probably is activated under PLCγ activity nearby Y1175 site gives rise to proliferation [234, 236]. After engagement of VEGF and VEGFR2, JAK2 phosphorylates STAT3, thus STAT3 plays its angiogenic role by upregulation of BCL2 and ANG2 gene expression [233].

As mentioned above, VEGF is the most important angiogenic factor in TME. Moreover, there are some factors that strengthen the VEGF/VEGFR axis. For example, bone morphogenetic protein (BMP) 2/6, increase the expression of VEGFR2, Dll4 and enhance the notch signaling pathway in the Tip cells. In contrast, it can be said that VEGF also increases the expression of BMP2/4 [237]. BMP9/10 also phosphorylate smad1/5 factors through BMP9/10-ALK1-endoglin signaling pathway, which upregulation of expression of id1 gene is the angiogenic activity of this pathway. In addition, it is important to note that increasing BMPs in cancers significantly increases resistance to conventional anti-VEGF therapies [238]. Erkasap et al. studies have proven that in most solid cancers, elevated levels of leptin as an adipokine and IL-1 have been associated with angiogenesis [239]. The binding of IL-1 to its receptor causes activation of IL-1 receptor kinase 1 (IRAK1) and IRAK4. IRAK1/4 causes recruitment of TNF receptor (TNFR)-associated factor-6 (TRAF6), ubiquitin conjugating enzyme 13 (Ubc13), and ubiquitin E2 variant 1a (Uev1a). Eventually, these factors activate TGF-β-activated kinase 1 (TAK1). TAK1, activity causes phosphorylation of MAP kinase kinase4/7 (MKK4/7), inhibitory kappa B kinase (IKK), and MKK3/6. These phosphorylated factors activate NF-κB, JNK, and p38 [240]. Yasmine F. Elesawy et al. have been reported that elevated leptin levels are directly related to MMP2/9 levels in cancers, and in addition, the induction of JNK,p38, and MAPK/ERK is another effect of leptin on angiogenesis in tumors [241].

According to studies, EphB4/ephrinB2 signaling pathways are involved in the migration of ECs and the formation of veins and arteries [242]. EphB4 and ephrinB2 are transmembrane receptor and ligand respectively which their connection brings on forward signaling in the cell that contains EphB4 and on the opposite side, reverse signaling occurs [243]. Forward signaling includes PI3K/Akt/NO pathway that stimulates PKG/Raf/Ras/MAPK and FAK pathways. These two pathways lead to proliferation and migration respectively. As well reverse signaling leads to the migration of ECs results from PAK/FAK signaling pathway [244]. Moreover, EphB4 directly phosphorylates STAT3 [243, 244] and STAT3 transmits the message of extracellular matrix (ECM) and pericytes into the nucleus that causes cell assembly [243, 245, 246].

Interestingly, in Wnt canonical signaling, β-catenin as a transcription factor is phosphorylated by glycogen synthase kinase 3β (GSK 3β), adenomatous polyposis coli (APC) and Axin. This phosphorylation brings degradation of β-catenin, but in contrast, when Wnt connects to FZD, APC/GSK-3β/Axin complex is inhibited [247, 248] and β-catenin translocates into the nucleus and expresses target genes along with LEF/TCF as co-transcriptional factors [249].

**Fig. 4:**
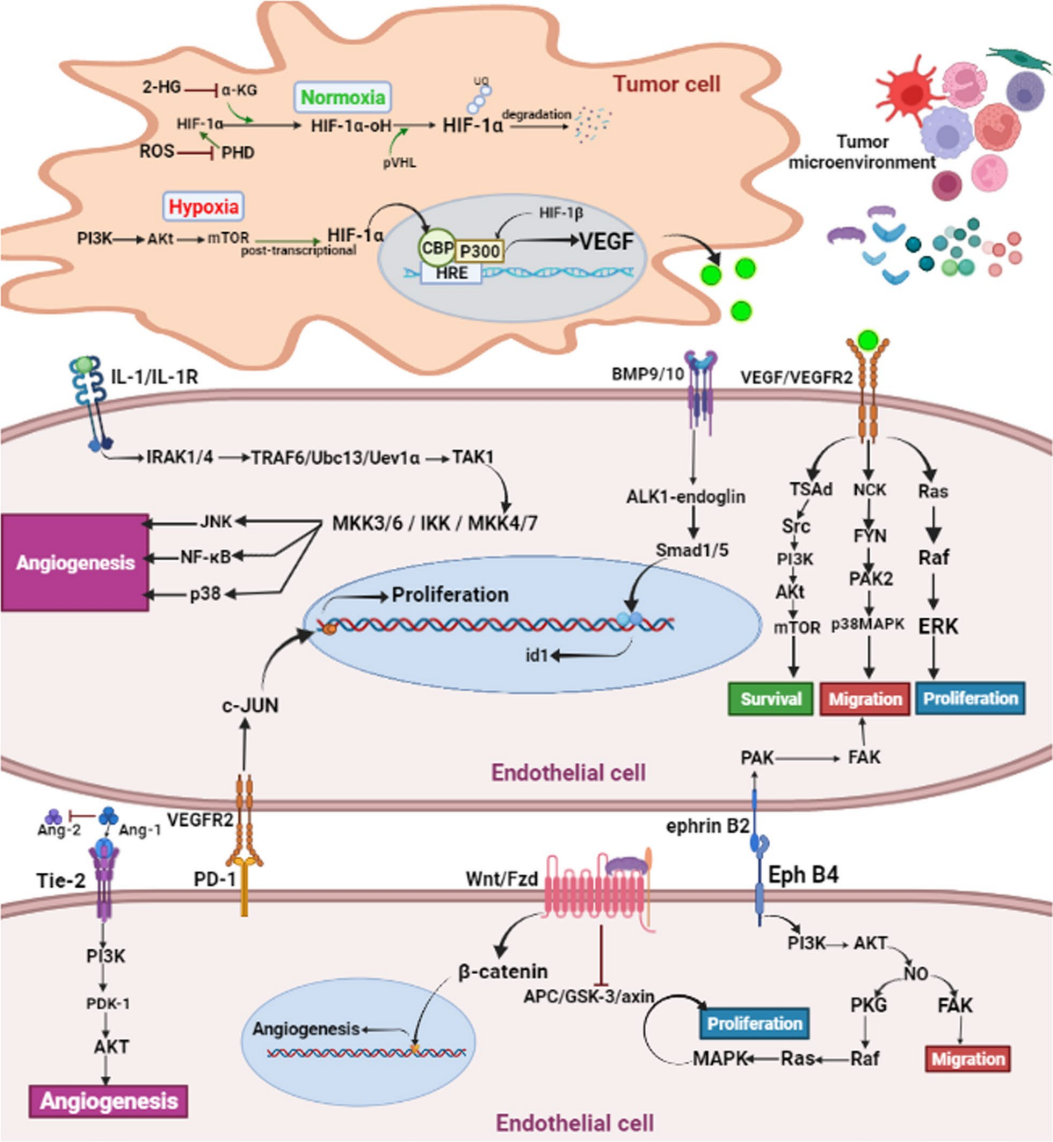
Illustration of EC signaling due to TME angiogenic activity and hypoxia-induced VEGF production in tumor cells: Hypoxia prevents HIF-1α from degradation in the proteasome and HIF-1α in the nucleus links with HIF-1β and CBP/P300 and upregulates VEGF gene transcription. Briefly, the most important pathways in EC lead to cell proliferation, survival, and migration that results in angiogenesis under the effect of soluble mediators secreted by TME cells. Proliferation: Ras/Raf/ERK related to VEGF/VEGFR2 signaling, and PI3K/AKT/MAPK related to EphB4/ephrinB2 forward signaling. Survival: TSAd/Akt/mTOR in VEGF/VEGFR2 signaling events, and AKT/PDK-1/Akt/mTOR in ANG-2/Tie-2 signaling events. Migration: NCK/ FYN/p38MAPK results from VEGF/VEGFR2 interaction, PI3K/AKT/FAK results from EphB4/ephrinB2 forward signaling, and PAK/FAK results from EphB4/ephrinB2 reverse signaling pathway

Aalso, Ang-1 and Ang-2 have the same receptor with the same extracellular site on their receptor and almost equal affinity. But the point is that connection of Ang-1 with Tie-2 as its receptor results PI3K/PDK-1/Akt signaling pathway while connection of Ang-2 with Tie-2 blocks this pathway and in the other word Ang-2 is an antagonist of Tie-2 [220, 250] [10, 50]. Survival and migration of epithelial cells are caused by PI3K/PDK-1/Akt/mTOR and PI3K/PDK-1/Akt/eNOS/NO signaling pathways [251, 252]. Obviously, increased expression of immune checkpoint ligands such as PD-L1 in TME is one of the immune escape mechanisms of tumor cells, but according to recent studies, increased PD-L1 in TME is directly related to angiogenesis. To that end, PD-L1 binds to VEGFR2 and actives C-JUN as a transcription factor in favor of the proliferation [253] (Fig. 4).

## Therapeutic perspectives and future direction

In the TME, a variety of metabolic processes and signaling molecules/pathways influence tumor angiogenesis. For the development of new therapeutic strategies, it is necessary to understand how these components function as angiogenic stimuli or as repressors. According to studies, anti-angiogenic drugs can reduce immunosuppressive cell numbers in TME and alleviate tumor-associated immunosuppression [254]. There are various challenges in cancer therapy. One of them is providing effective therapies while minimizing side effects [255]. So, patients may benefit from a variety of combinations of immune checkpoint blockers (ICBs). Recent studies revealed that there are multiple adverse events (AEs) and immune-related AEs (irAEs) due to the utilization of ICIs in cancer patients [256]. In addition, since cancer and coronavirus disease 2019 (COVID-19) have very different immune systems, ICI may cause some unpredictable irAEs in cancer patients with COVID-19 infection [257]. As a way to safely treat cancer patients and prevent immune checkpoint blockade-induced toxicities and autoimmunity, we can use anti-angiogenic drugs solely or combined with ICBs to enhance the safety and effectiveness of therapy for these patients. Transcriptional factors hypoxia-inducible factors (HIFs) regulate gene expression, phenotypic and metabolic changes including tumor angiogenesis, metastasis, and invasion, during hypoxic tumor response [258, 259]. Thus, directly targeting HIF-1 or its indirectly targeting by agents inhibiting its up and down-stream signaling/factors, such as translational inhibitor and microtubule-targeting metabolite 2-methoxyestradiol (2ME2), heat shock protein inhibitors, histone deacetylase inhibitors, and even topotecan (a topoisomerase I inhibitor), are potential strategies for cancer therapy. Moreover, hypoxia conditions in cancerous cells and TME represent an opportunity to develop hypoxia pro-drugs such as evofosfamide and gemcitabine activated via redox under extremely hypoxic conditions. As mentioned previously, carbonic anhydrase enzymes are upregulated via hypoxia and hyper-activation of HIF-1. To prevent the high activity of carbonic anhydrase enzymes, Girentuximab, an anti-carbonic anhydrase enzyme (CAIX) antibody, was developed to stimulate both innate and adaptive immune-mediated killing of tumor cells. Other drugs, directly and indirectly, targeting HIF-1 are 2ME2, 17-AAG, Vorinostat, PT2977, EZN-2208, and CRLX101 whose detailed properties are described in Table 1 [260–264].

**Table 1:** Drugs for cancer treatment with targeting signaling pathways and factors associated with tumor angiogenesis

Drug	Target	Cancer/Cell line	Ref
Topotecan (Hycamtin)	Topoisomerase 1 inhibitor	Several cancer types such as ovarian, cervical and lung cancers	[261, 262]
Evofosfamide	Cross-linking with DNA as alkylating agent in hypoxia condition	Phase III clinical trials in patients with pancreatic adenocarcinoma and murine prostate cancer model	[262, 263]
Gemcitabine	A cytidine analog	Several human cancer types including pancreatic adenocarcinoma, lung, breast and ovarian cancers	[263]
Girentuximab (Rencarex)	Anti-carbonic anhydrase enzyme (CAIX) antibody	Several human cancer types including RCC	[264, 281]
2ME2	HIF-1 inhibitor	Several human cancer types including RCC	[261, 262]
17-AAG	HIF-1 inhibitor	Several human cancer types including melanoma	[262]
Vorinostat	HIF-1 inhibitor	head and neck squamous cell carcinoma	[262]
PT2977	HIF-1 inhibitor	Plus, cabozantinib in advanced RCC	[262, 282, 283]
EZN-2208	HIF-1 inhibitor	Metastatic CRC	[284]
CRLX101	HIF-1 inhibitor	Prostate cancer Plus, bevacizumab in smetastatic RCC	[285–287]
Cetuximab	VEGFR supressor	Metastatic CRC	[288]
Cediranib	VEGFR supressor	Glioblastoma multiforme (GBM)	[289]
Tivozanib	VEGFR supressor	GBM	[290]
Axitinib	VEGFR supressor	RCC	[291]
Pazopanib	VEGFR, c-KIT and PDGFR tyrosine kinases supressor	advanced or metastatic RCC	[292]
Sunitinib	VEGFR supressor	advanced or metastatic RCC	[293]
Sorafenib	VEGFR supressor	HCC	[294]
Bevacizumab (avastin)	VEGFA inhibitor	Lung cancer, breast cancer and CRC	[295]
Durvalumab	PD-L1 supressor	Ovarian, endometrial and TNBC cancers	[296]
Atezolizumab	PD-L1 supressor	Metastatic nonsquamous NSCLC. Advanced triple-negative breast cancer	[297–299]
Rebastinib	Tie2 receptor supressor	Metastatic mammary carcinoma and pancreatic neuroendocrine tumors	[276]
Trebananib (AMG386)	Tie2 receptor suppresor	Advanced solid tumors	[278]
Vantictumab and OTSA101	Frizzled inhibitors	Several cancer types such as colorectal and gastric cancers	[273, 274]
CGX1321 and Ipafricept	Wnt inhibitors	Metastatic solid tumors such as HCC	[275, 300]
HuMax-IL8 (BMS-986253)	anti-IL-8 monoclonal antibody	chordoma, colorectal, prostate, ovarian, papillary thyroid, chondrosarcoma, and esophageal cancers	[279]
GLPG1790	Ephrin receptor inhibitor	Breast cancer, CRC	[301, 302]

Tumor development is closely associated with angiogenesis, and VEGFR2 plays a crucial role in tumor angiogenesis. There is extensive VEGFR2 expression in the blood vessels, especially in tumor microvessels. As well, VEGFR2 is found on the surface of a variety of immune cells, including macrophages, DCs, and Tregs [142]. Therefore, drugs directly/indirectly inhibiting VEGFR2 activity may be a potential anti-angiogenic therapy for different solid tumors. For instance, cetuximab, tivozanib, cediranib and Tyrosine kinase inhibitors (TKIs) like axitinib, sorafenib, sunitinib and pazopanib. Besides VEGFR-1, 2 and 3 inhibitions, Pazopanib also, suppresses c-KIT and platelet-derived growth factor receptor (PDGFR) tyrosine kinases (TKs). Moreover, Bevacizumab (also called avastin) is an inhibitor of VEGFA in patients with lung cancer and colorectal cancer (CRC) [265–268]. Programmed death-1 receptor (PD-1) is a checkpoint mediator acting primarily in the priming phase of immune responses, that interaction with its ligand, programmed death-ligand 1 (PD-L1), promotes the immunosuppressive state in the TME. PD-1 or PD-L1 blockade can thus offer promising therapeutic options for patients with advanced cancers. In this line, durvalumab, as a PD-L1 inhibitor, showed a promising response rate in patients with ovarian, endometrial and triple-negative breast cancer (TNBC) when it was combined with cediranib. Also, it was shown that combination therapy of bevacizumab with atezolizumab, another PD-L1 inhibitor, created an immunogenic microenvironment for tumor regression [269–271]. Also, Ang/Tie2 receptor axis stimulates angiogenic signaling, and studies suggest Ang-1, 2 and Tie2 receptor as an anti-angiogenic target for therapy [272]. Due to the researches, it has been described an angiogenic function for Wnt/FZD signaling pathway [273] via inducing VEGF upregulation, which leads to unstable and leaky tumor angiogenesis [273, 274]. Thus anti-FZD drugs such as Vantictumab and OTSA101 or anti-Wnt drugs such as CGX1321 and Ipafricept may have anti-angiogenic effects [274, 275].

Rebastinib is one of the Tie2 receptor inhibitors with picomolar potency increasing tumor growth, angiogenesis, and metastasis in metastatic mammary carcinoma and pancreatic neuroendocrine tumors [276]. A combinatorial approach targeting Dll4/Notch and EphB2/EphB4 may also lead to disrupting tumor angiogenesis [277]. Trebananib (AMG386) is a selective antagonist peptide-Fc fusion protein inhibiting the interaction between Ang-1, Ang-2 and Tie2 suppressing the endothelial cell proliferation and subsequently, tumor growth [278]. Angiogenesis and immunosuppressive cell recruitment can also be increased by IL-8. Researchers found that targeting IL-8 or IL-8R, such as HuMax-IL8 (BMS-986253) as an anti-IL-8 monoclonal antibody [279], would be able to provide anti-tumor and anti-angiogenic responses (Table 1) [280]. Despite the fact that metabolic processes and signaling pathways associated with tumor angiogenesis are still not fully known, this review opens new windows of therapeutic insight into intervention for the treatment of cancer patients. Therefore, we encourage researchers to target TME cells and their mediators (Fig. 1), metabolic profiles (Figs. 2, 3), and intracellular signaling pathways (Fig. 4) in order to inhibit angiogenesis in solid tumors.

## Conclusion

Nowadays, cancer therapy has been revolutionized by Immune checkpoint blockers (ICBs). While, a large number of patients fail to respond to the ICBs, or suffer a relapse with long-term toxicity (i.e., autoimmunity). The polarized TME is crucial in the outcome of the patient response to an ICB, therefore theoretically treating a vascularized TME could improve the effectiveness of these therapies. We suggest using drugs that inhibit angiogenic signaling pathways stimulated due to metabolic processes or employing drugs that eliminate the tumor or tumor-associated cells that contribute to tumor angiogenesis and invasion. Also, it can combine with other cancer therapy approaches such as ICBs.

## Data Availability

Not applicable.

## Abbreviations

TME: Tumor microenvironment
ECM: Extracellular matrix
CAF: Cancer-associated fibroblast
TAM: Tumor-associated macrophage
TAN: Tumor-associated neutrophil
MDSC: Myeloid-derived suppressor cells
NK cells: Natural killer cells
DC: Dendritic cell
TEM: Tie2-expressing monocytes
ILCs: Innate lymphoid cells
Treg: Regulatory T cell
ICBs: Immune checkpoint blockers
PI3K: Phosphatidylinositol 3-kinase
HIF: Hypoxia-inducible factor
mTOR: Mammalian target of rapamycin
LDHA: Lactate dehydrogenase A
LDHB: Lactate dehydrogenase B
MCT: Monocarboxylate transporter
ACSS2: Acetyl-CoA synthetase 2
ACLY: ATP citrate lyase
OXCT: Succinyl-CoA:3-oxoacid-CoA transferase
BDH: D-βOHB dehydrogenase
GDH: Glutamate dehydrogenase
GLS: Glutaminase
GS: Glutamine synthetase
OAA: Oxaloacetic acid
ECM: Extracellular matrix
MCT1: Monocarboxylate transporter 1
MMPs: Matrix metalloproteinases
Treg: Regulatory T cells
HA: Hyaluronic acid
CAIX: Carbonic anhydrase IX
IDO1: Indoleamine-2, 3-dioxygenase 1
TDO2: Tryptophan-2, 3-dioxygenase 2
Kyn: Kynurenine
AhR: Aryl—hydrocarbon receptor
PDGFR: Platelet-derived growth factor receptor
PD-L1: Programmed death-ligand 1
TKIs: Tyrosine kinase inhibitors
CRC: Colorectal cancer
TNBC: Triple-negative breast cancer
RCC: Renal cell carcinoma
HCC: Hepatocellular carcinoma
NSCLC: Non-small cell lung cancer
